# *Big Data*, inteligencia artificial y medicina de laboratorio: la hora de la integración

**DOI:** 10.1515/almed-2021-0014

**Published:** 2021-02-19

**Authors:** Damien Gruson

**Affiliations:** Pôle de recherche en Endocrinologie, Diabète et Nutrition, Institut de Recherche Expérimentale et Clinique, Cliniques Universitaires St-Luc and Université Catholique de Louvain, Bruxelles, Belgium; Department of Clinical Biochemistry, Cliniques Universitaires St-Luc and Université Catholique de Louvain, Bruxelles, Belgium

**Keywords:** aprendizaje automático, *Big Data*, cáncer, cardiovascular, ética, inteligencia artificial

En el campo de la medicina de laboratorio, se están integrando constantemente innovaciones tecnológicas, que facilitan las decisiones clínicas, el seguimiento de las enfermedades y la seguridad del paciente [1]. La innovación posee un enorme potencial a la hora de transformar los sistemas sanitarios, así como la medicina de laboratorio, proporcionando al personal sanitario la información y herramientas necesarias para optimizar la calidad de sus servicios, a su vez que se mejoran los resultados clínicos, empleando menos recursos [2]. En la innovación también se incluye el potencial de los datos y la Inteligencia Artificial (IA). Actualmente, la ciencia de datos y la IA están revolucionando la vida diaria de los ciudadanos, así como el funcionamiento de las instituciones, las ciudades y los intercambios comerciales. La IA es un término muy amplio, que combina la informática con sofisticados modelos matemáticos, lo que permite el desarrollo de complejos algoritmos capaces de simular la inteligencia humana en tareas tales como la resolución de problemas y el aprendizaje [2]. Una de las aplicaciones más prometedoras del *Big Data* y la IA es en los servicios sanitarios, donde la IA posee un gran potencial, que revolucionará los actuales protocolos de diagnóstico, así como la prevención y el control de enfermedades, mejorando notablemente la seguridad del paciente, así como la calidad asistencial [3]. La cada vez mayor accesibilidad a grandes volúmenes de datos (a través de los historiales médicos electrónicos, los sistemas informáticos de los laboratorios, las llamadas ciencias *ómicas*, las aplicaciones digitales, etc.) está suscitando grandes expectativas en el campo del *Big Data* y la IA.

La pandemia de Covid-19 ha acelerado dicho crecimiento. Sin descontar los efectos devastadores que la Covid-19 está teniendo en la población en todo el mundo, esta pandemia también ha servido como catalizador para la innovación y la aplicación de la IA. El principio de adaptación al virus nos ha llevado a modificar nuestro entorno tecnológico, así como las prácticas existentes, con el fin de hacer frente a una cada vez mayor demanda de una asistencia de calidad, aún con unos recursos sanitarios limitados [2]. Recientemente, se ha publicado una opinión del Panel de Expertos de la Comisión Europea sobre Formas Efectivas de Invertir en Salud sobre la organización de unos servicios socio-sanitarios resistentes tras la pandemia de la COVID-19 [4]. Esta opinión identifica los pilares de unos servicios socio-sanitarios resistentes y con capacidad de adaptación, examina los elementos y condiciones necesarios para la construcción de capacidades para fortalecer la capacidad de resistencia de los sistemas sanitarios, aborda las necesidades sanitarias de los pacientes más vulnerables en períodos de crisis, y define un proyecto para comprobar la capacidad de adaptación de los sistemas sanitarios. Entre los diferentes elementos abordados, se hace especial hincapié en el poder de los datos, así como en el valor de la integración de datos y de la IA a la hora de afrontar brotes inesperados [4]. Ya se han hecho públicas varias formas de aplicar la IA a la Covid-19, como es el caso de las aplicaciones de rastreo de brotes basadas en IA, el análisis de publicaciones científicas basadas en IA y el triaje empleando el procesamiento de lenguaje natural para identificar a posibles pacientes, así como herramientas pronósticas basadas en TAC para gestionar las capacidades del sistema [2].

Actualmente, existen multitud de ejemplos del valor añadido que el *Big Data* y la IA pueden aportar en la gestión de enfermedades crónicas no infecciosas como el cáncer o las enfermedades cardiovasculares. Las herramientas de IA aumentan la eficacia de los profesionales médicos, así como la precisión en las decisiones clínicas, permitiendo así mejorar la calidad asistencial, así como la seguridad del paciente. El *Big Data* y la IA pueden facilitar la toma de decisiones, mejorando el rendimiento diagnóstico y pronóstico. En las áreas de oncología y medicina cardiovascular, ya se han obtenido resultados prometedores sobre la aplicación de la IA en la extracción de información fenotípica detallada, a partir de pruebas de imagen y datos analíticos, así como de otros dispositivos médicos [2], [ 3]. La IA puede ayudar a identificar nuevos genotipos y fenotipos relacionados con enfermedades crónicas, permitiendo así mejorar la calidad asistencial y la rentabilidad, a su vez que se reducen los índices de readmisión y mortalidad [5]. Las tecnologías basadas en la IA también pueden ofrecer orientación terapéutica y mostrar la posible toxicidad de las terapias, así como predecir las probabilidades de fracaso en los ensayos clínicos y los efectos secundarios de las combinaciones de fármacos. Además, las sofisticadas herramientas basadas en IA, como el uso de gemelos numéricos o *“digital twins”*, permiten a los médicos anticipar la respuesta de un paciente a una enfermedad o fármaco, ayudando a los equipos médicos a preparar los ensayos clínicos seleccionando a los pacientes adecuados para cada ensayo [2].

El *Big Data* y la IA facilitarán el desarrollo de una medicina personalizada mediante la predicción temprana de riesgos, prevención e intervención terapéutica. Los datos analíticos y biológicos contribuirán a optimizar la eficiencia y calidad de las herramientas basadas en la IA. Además del impacto clínico, también cabe mencionar el potencial del *Big Data* y la IA a la hora de mejorar la eficiencia y sostenibilidad de los laboratorios, identificando áreas de desaprovechamiento, mejorando los procesos y racionalizando la solicitud de pruebas analíticas [6].

Dado que el *Big Data* y la IA ofrecen un valor y unas ventajas específicas, es importante que su uso se integre adecuadamente en la práctica clínica. No obstante, aún quedan algunas dificultades por solventar, que se muestran en la Tabla 1 [2]. Se debe dar prioridad a la construcción de ecosistemas e infraestructuras de datos que fomenten y conformen la aplicación de la IA, así como integrar y aprovechar el futuro “Espacio Europeo de Datos”, implicar al personal sanitario en la validación externa y probar la transferibilidad de las aplicaciones de IA, incorporar contenidos digitales y relacionados con la IA en los currículos formativos de los profesionales sanitarios, desarrollar un marco legal y ético sólido, basado en un enfoque de la IA basado en los riesgos y centrado en el paciente [2], [ 5]. Los especialistas de medicina de laboratorio, así como los laboratorios clínicos jugarán un papel fundamental en esta evolución, y su misión será proporcionar datos estructurados y armonizados, proporcionando orientación sobre el tipo de datos a emplear, integrando equipos multidisciplinares para la validación de las herramientas basadas en IA y empleando herramientas para la optimización de la seguridad del paciente, los procesos analíticos y los flujos de trabajo.

**Tabla 1:** Retos del *Big Data* y la IA [2].

1.	Establecer un marco legal completo que regule la aplicación de la IA y actualizar la normativa correspondiente, para garantizar que esta contempla el uso de la IA.
2.	Identificar y promover buenas prácticas con el fin de garantizar la solidez de los sistemas de *Big Data* e IA en el sector de la salud, tanto en las fases de desarrollo como de uso, con el fin de reducir posibles sesgos y errores en la toma de decisiones basada en la IA.
3.	Acelerar el desarrollo de un Espacio Común Europeo de Datos Sanitarios como parte de una estrategia general orientada a resolver la actual fragmentación de los datos sanitarios de la UE.
4.	Mejorar la interoperatividad y promover el desarrollo de infraestructuras de datos, con el objeto de proporcionar un flujo de datos estandarizado y fiable, regulado por las correspondientes normativas en materia de ciberseguridad y protección de datos.
5.	Apoyar el desarrollo de un sistema nacional de historias médicas electrónicas y mejorar la interoperatividad de los datos sanitarios.
6.	Dotar a los profesionales sanitarios de las habilidades y conocimientos necesarios para maximizar la aplicación de la IA, y realizar una revisión general de la normativa relativa a la formación de los distintos profesionales sanitarios, para determinar si esta es la adecuada para facilitar la provisión de servicios sanitarios basada en la IA centrada en el paciente.
7.	Garantizar que la IA se aplica respetando las leyes europeas de protección de datos, a su vez que se logra un equilibro entre los avances científicos y la protección de los pacientes.
8.	Invertir en investigación e innovación para promover la actualización de las aplicaciones sanitarias basadas en IA y fomentar el acceso de los pacientes a las mejores tecnologías actualmente disponibles.
9.	Aplicar una serie de mecanismos para garantizar la formación de los pacientes, con el fin de que estos puedan conocer y emplear la IA, permitiéndoles así participar activamente en la gestión de su salud.

En conclusión, la ciencia de datos, el *Big Data* y la IA, en sus diferentes aplicaciones, permitirán revisar la experiencia de los pacientes en todo el circuito asistencial, desde la prevención hasta el despistaje y el diagnóstico temprano, pasando por el tratamiento y el manejo de las enfermedades. Los especialistas de medicina de laboratorio y los laboratorios clínicos serán fundamentales a la hora de lograr una integración eficiente y segura de las herramientas basadas en *Big Data* e IA, así como de formar al personal sanitario y a los pacientes. Con una integración adecuada, el *Big Data* y las tecnologías de IA tendrán un importante impacto económico en los sistemas sanitarios europeos.
